# A dynamic transmission model for predicting trends in Helicobacter pylori and associated diseases in the United States.

**DOI:** 10.3201/eid0603.000302

**Published:** 2000

**Authors:** M F Rupnow, R D Shachter, D K Owens, J Parsonnet

**Affiliations:** Stanford University, Stanford, California, USA. marcia.rupnow@stanfordalumni.org

## Abstract

To assess the benefits of intervention programs against Helicobacter pylori infection, we estimated the baseline curves of its incidence and prevalence. We developed a mathematical (compartmental) model of the intrinsic dynamics of H. pylori, which represents the natural history of infection and disease progression. Our model divided the population according to age, infection status, and clinical state. Case-patients were followed from birth to death. A proportion of the population acquired H. pylori infection and became ill with gastritis, duodenal ulcer, chronic atrophic gastritis, or gastric cancer. We simulated the change in transmissibility consistent with the incidence of gastric cancer and duodenal ulcer over time, as well as current H. pylori prevalence. In the United States, transmissibility of H. pylori has decreased to values so low that, should this trend continue, the organism will disappear from the population without targeted intervention; this process, however, will take more than a century.


### A Dynamic Transmission Model for Predicting Trends in *Helicobacter pylori* and Associated Diseases in the United States

Marcia F.T. Rupnow,* Ross D. Shachter,* Douglas K. Owens,*† and Julie Parsonnet*
*Stanford University, Stanford, California, USA; †Department of Veterans Affairs Health Care System, Palo Alto, California, USA

To assess the benefits of intervention programs against ***Helicobacter pylori*** infection, we estimated the baseline curves of its incidence and prevalence. We developed a mathematical (compartmental) model of the intrinsic dynamics of ***H. pylori***, which represents the natural history of infection and disease progression. Our model divided the population according to age, infection status, and clinical state. Case-patients were followed from birth to death. A proportion of the population acquired ***H. pylori*** infection and became ill with gastritis, duodenal ulcer, chronic atrophic gastritis, or gastric cancer. We simulated the change in transmissibility consistent with the incidence of gastric cancer and duodenal ulcer over time, as well as current ***H. pylori*** prevalence. In the United States, transmissibility of ***H. pylori*** has decreased to values so low that, should this trend continue, the organism will disappear from the population without targeted intervention; this process, however, will take more than a century.

***Helicobacter pylori***, a common human bacterial pathogen (1), causes peptic ulcer disease, gastric cancer, and gastric mucosa-associated lymphoid tissue lymphoma. Current U.S. guidelines from the National Institutes of Health recommend antimicrobial treatment only for ***H. pylori*** patients with peptic ulcer disease (2). Because asymptomatic infection is very common, treatment of all asymptomatic persons would be expensive and might promote antibiotic resistance. Prophylactic and therapeutic vaccines against ***H. pylori*** are being developed (3,4); however, if vaccines are to be cost-effective, companies must take into account the changing epidemiology of ***H. pylori*** and related diseases. Knowledge of these trends can also allow health agencies to predict resource allocations needed for these diseases.

In industrialized countries, ***H. pylori*** incidence has been decreasing in successive generations, without any targeted intervention (5-7). Quantifying the benefits of intervention in reducing disease incidence or cost requires an analytical model that estimates the natural course of ***H. pylori*** and associated diseases. The model could then estimate the decrease in ***H. pylori*** incidence that would result from an intervention strategy, relative to the natural course of infection.

We present an analytical framework to model ***H. pylori*** transmission dynamics and its subsequent disease progression. Our goal is to estimate future trends of ***H. pylori*** and associated diseases in the United States based solely on its natural history (i.e., without intervention).

### Methods

### Model Description and Epidemiologic Assumptions

Our analysis is based on a dynamic compartmental model, a technique in which the population is divided into compartments and mathematical equations describe the transfer of persons from one compartment to another (8). In this technique, the incidence of ***H. pylori*** infection

Address for correspondence: Marcia F.T. Rupnow, Health Research and Policy T221, Stanford, CA 94305-5405, USA; fax: 650-725-6951; e-mail: marcia.rupnow@stanfordalumni.org.

is calculated as a function of the number of susceptible and infected persons in the population and a constant called the transmission parameter (*ß*). We sought transmission parameters that would best explain historical trends and allow estimates of future trends of ***H. pylori*** prevalence and the incidence of ***H. pylori***–associated gastric cancer (GC) and duodenal ulcer (DU).

We developed a compartmental model that captures the age-dependence of ***H. pylori*** infection and disease progression in infected persons (Figure 1)(6,9-17). The population was compartmentalized according to three factors:

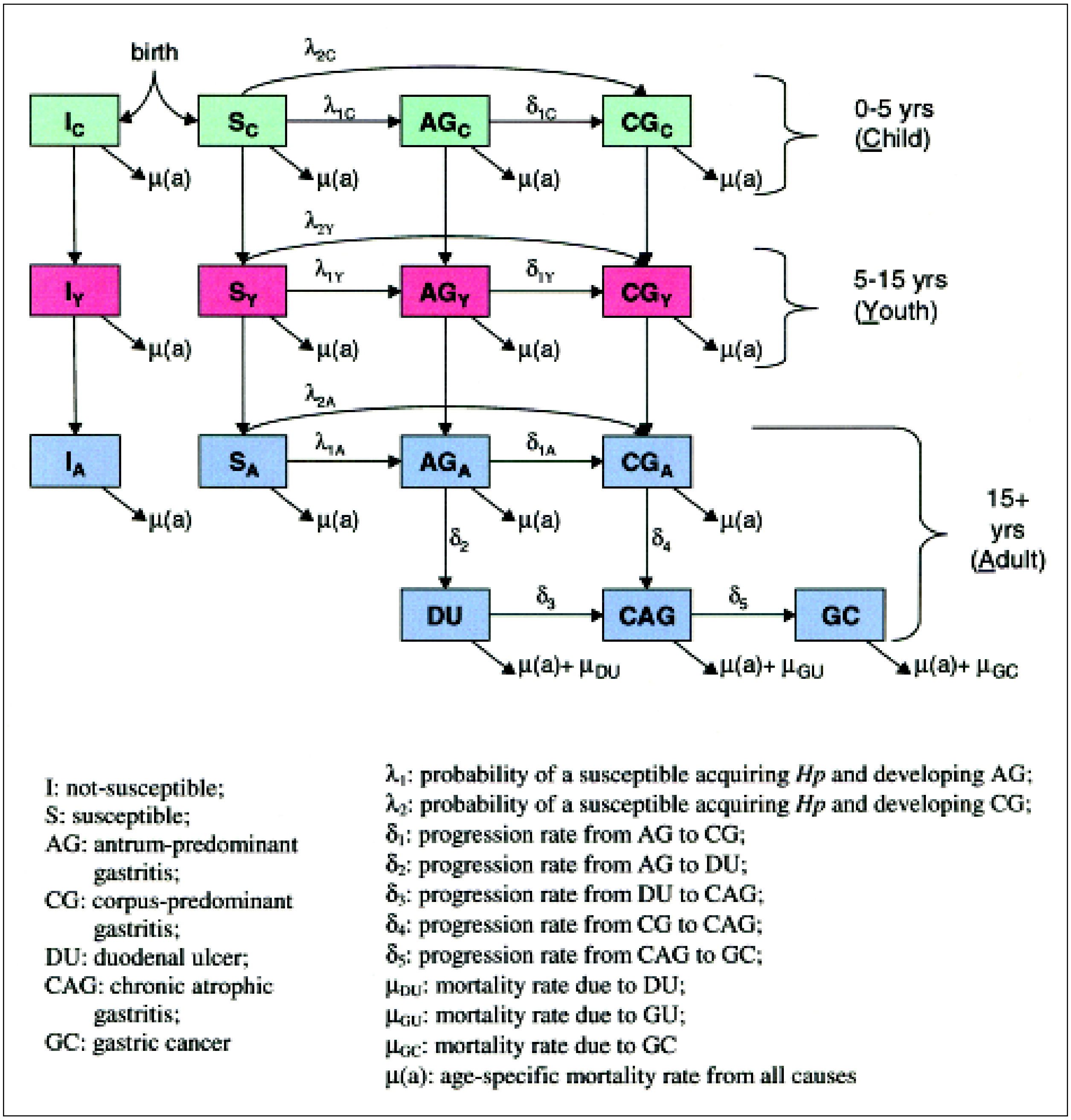

Figure 1. Compartmental model of ***Helicobacter pylori*** transmission and disease progression. In this model, the population is divided into compartments according to age, infection status, and clinical state. Boxes represent population subgroups and arrows indicate transitions between subgroups, as well as flow into and out of the population (birth and death).

age (child [4 years old], youth [5 to 14 years old], and adult [>15 years old]); infection state (uninfected and infected); and clinical state (normal stomach, antrum-predominant gastritis, corpus-predominant gastritis, DU, chronic atrophic gastritis [CAG], and GC) (Appendix). DU and GC states corresponded to persons whose illnesses were caused by ***H. pylori*** infection (***H. pylori***–associated DU and GC). We excluded from our model DU and GC not related to ***H. pylori***. Computer simulation was used to solve the system of equations numerically and calculate the number of persons in each compartment over time.

We modeled the possibility of being born susceptible or not susceptible to ***H. pylori*** (Figure 1). The nonsusceptible or "isolated" group comprised persons who could not become infected for physiologic, physical, or immunologic reasons. This conforms with epidemiologic studies indicating that, even within populations at high risk, a small percentage (10% to 20%) does not become infected with ***H. pylori*** (9,10).

A susceptible person could become infected with ***H. pylori*** at any age and either antrum- or corpus-predominant gastritis could develop. The model represents the net flow from one clinical state to the other. Thus, we represented a positive flow from antrum gastritis to corpus gastritis because this progression is much larger than the reverse. From the gastritis states, we modeled the possibility of further clinical progression to DU, CAG, and GC. The model is consistent with previously reported outcomes in that persons with antrum-predominant gastritis are at higher risk for DU, while those who have corpus-predominant gastritis are at higher risk for CAG, GU, and GC (11,12-17). We also incorporated the possibility of CAG and GC developing in DU patients.

### Input Data and Sources

Input assumptions are divided into disease and population parameters (Table). We adopted a constant demographic characteristic of birth and death rates, to eliminate complexities associated

Table. Input variables and sources

| Symbol | Description | Value | Explanation | Source |
|---|---|---|---|---|
| **Population parameters** | | | | |
| $\boldsymbol{\mu}$_(a) | Death rate (per person per year) | Age-specific | | 18 |
| Z | Population size (persons) | 200,000 | | Assumption |
| ***II*** | Birth rate (persons per year) | 2,962 | Derived from disease-free equilibrium simulation | |
| $p_I$ | Proportion (%) nonsusceptible | 20 | Based on data from developing countries | Assumption |
| **Disease parameters** | | | | |
| $p_C$ | Proportion (%) of children with antrum (vs. corpus) gastritis | 5 | | Assumption |
| $p_Y$ | Proportion (%) of youths with antrum (vs. corpus) gastritis | 75 | Based on simulation | Assumption |
| $p_A$ | Proportion (%) of adults with antrum (vs. corpus) gastritis | 95 | | Assumption |
| $\delta_{1C}$ | $AG_C$ to $CG_C$ (per person per year) | 0 | | Assumption |
| ___$\delta_{1Y}$ | $AG_Y$ to $CG_Y$ (per person per year) | 0 | | Assumption |
| ___$\delta_{1A}$ | $AG_A$ to $CG_A$ (per person per year) | 0.0010 | 1% progression in 10 yrs | Assumption |
| $\delta_2$ | $AG_A$ to DU (per person per year) | 0.0046 | 14% progression in 32 yrs | 22 |
| ___$\delta_3$ | DU to CAG (per person per year) | 0.0020 | 2% progression in 10 yrs | 15 |
| ___$\delta_4$ | $CG_A$ to CAG (per person per year) | 0.0112 | 30% progression in 32 yrs | 22 |
| ___$\delta_5$ | CAG to GC (per person per year) | 0.0030 | 3% progression in 10 yrs | 13 |
| ___$\mu_{DU}$ | Mortality rate due to DU (per person per year) | 0.0050 | | 23 |
| ___$\mu_{GU}$ | Mortality rate due to GU (per person per year) | 0.0100 | | 24 |
| ___$\mu_{GC}$ | Mortality rate due to GC (per person per year) | 0.3219 | 80% death in 5 yrs | 25 |

GC = Gastric cancer; DU = Duodenal ulcer; CG = Corpus-predominant gastritis; AG = Antrum-predominant gastritis; CAG = Chronic atrophic gastritis.

with changing demographics (8). Baseline demographics were characteristic of the general U.S. population in 1950. The age-specific death rates from all causes were derived from the survival curve of the Vital Statistics Report (18). We assumed an initial population of 200,000, which corresponds to the population of a medium-sized city in the United States (19). The birth rate was set so that the size of the population remained constant when ***H. pylori***–associated diseases were not present. This birth rate was derived from a disease-free simulation exercise in which we assumed zero infection with ***H. pylori***.

The proportion of isolated (nonsusceptible) persons in the population, $p_I$, is an assumption based on data from developing countries, in which the prevalence of ***H. pylori*** among adults achieves an asymptote of approximately 80% by age 30 (1,20). We assumed that earlier acquisition of ***H. pylori*** is associated with development of corpus-predominant gastritis and subsequent CAG, GU, and GC, and that acquisition at older ages is more likely to lead to antrum-predominant gastritis and subsequent DU (11,12,21). Therefore, we adopted 5% and 95% for the proportions of gastritis initially restricted to the antrum in children and adults, respectively. We chose 75% as the proportion of antrum gastritis in youths; this estimation had to be closer to that of adults than to that of children for the delay of ***Hp*** acquisition to result in more persons with antrum-predominant gastritis and subsequent DU.

We derived the rates of clinical progression from gastritis to cancer from published information (13,15,22-25). The model also incorporated deaths from DU, GU, and GC. GU is not an explicit compartment in our model; instead, we modeled the possibility that a person with CAG would die of GU, in accordance with previous studies that documented an increased risk for GU in persons with CAG (12,16).

### Transmission Parameters, Validation of the Model, and Future Trends

We calculated incidence as a function of the number of susceptible and infected persons in the population and the transmission parameter, ***ß***, which is a constant that characterizes infectivity of the pathogen. The number of infected persons at each period was determined by adding the number of persons with antrum- and corpus-predominant gastritis, DU, and CAG. We multiplied the number of CAG patients by a factor (<1), to reflect the fact that the infection may subside (or ***H. pylori*** density may be lower) as a result of atrophy (26), and therefore would contribute less to the transmission of ***H. pylori***. Furthermore, we assumed that the role of GC patients as a source of infection was negligible.

***ß*** may differ according to the population, age of infective and susceptible persons, environmental conditions, household sanitary and hygiene practices, and genetic characteristics of the organism itself (e.g., strain differences). Direct measurement of ***ß*** is not possible for most infections (8). However, the value of ***ß*** and its change over time must be estimated to assess the impact of public health programs against ***H. pylori***. Since the incidence and prevalence of ***H. pylori*** have been decreasing in the absence of a vaccine (which would decrease the number of susceptible persons) or widespread eradication therapies (which would decrease the number of infected persons), the historical decrease in ***H. pylori*** incidence must represent a decrease in ***ß***. Thus, we estimated the value of ***ß*** in the 19th century by simulating an endemic equilibrium condition of ***H. pylori*** in the U.S. population. We then estimated the change in ***ß*** that would account for the observed patterns of DU and GC. Finally, we extrapolated ***ß*** for the 21st century to estimate the future trends in ***H. pylori***–associated DU and GC.

We set up the model with nine transmission parameters, according to the age groups of infected and susceptible persons. To find ***ßs*** in mid-1800s, we assumed that the pattern of infection in the United States at that time was similar to that in developing countries today, i.e., rapid acquisition of ***H. pylori*** at younger ages (up to 5 years old) and subsequent slower acquisition rate, achieving a maximum prevalence of approximately 80%. In personal interviews, four experts in ***H. pylori*** epidemiology provided an assessment of the transmissibility among different age groups; they estimated transmission among children to be 5 to 10 times higher than among adults (Hazell SL, Megraud F, Blaser M, Correa P, unpub. comm.). No assessment could be obtained for the different inter-age group transmissions ($ß_{CY}$, $ß_{CA}$, $ß_{YC}$, $ß_{YA}$, $ß_{AC}$, $ß_{AY}$). For the baseline analysis, we assumed that inter-age group transmissions are negligible compared with intra-age group transmissions ($ß_{CC}$, $ß_{YY}$, $ß_{AA}$) because on average, interaction among persons of the same age group is the highest.

Once we established transmission parameters for the mid-1800s, we estimated changes in transmissibility that could explain the historical patterns of GC and DU. GC incidence and deaths have been decreasing since the beginning of the 20th century (25,27,28). DU, however, is characterized by a rise and fall within the 19th century (29-31). For DU, long-term estimates were based on other statistics, such as deaths, physician visits, and hospitalized patients.

Investigators have postulated that the different patterns of GC and DU could be explained by a more rapid decline in childhood than in adult incidence, thereby decreasing the proportion of new infections acquired in childhood (10,32). We evaluated whether changes in $\beta$ consistent with this hypothesis could reasonably explain disease patterns. We then extrapolated the transmissibility values into the year 2100 on the basis of our projections for future socioeconomic changes that would affect the dynamics of ***H. pylori*** transmission, and estimated the future trends in ***H. pylori*** prevalence, as well as the incidence of ***H. pylori*–**associated GC and DU.

### Model Implementation

We implemented the model using the software package Powersim Constructor Version 2.5 (Powersim Corp., Herndon, VA), integrated with Microsoft Excel 97 (Microsoft Corp., Redmond, VA). We set Powersim to use Runge-Kutta 4th-order integration method and time-step of 1 year.

To obtain correct flows of age groups, at the implementation level we further subdivided the child and youth groups by 1-year age increments (i.e., birth through first birthday, 1 year of age through second birthday . . . 14 years of age through 15th birthday). We subdivided the adult compartments by 5-year age increments up to 85 years of age (15 years of age through 20th birthday . . . 80 years of age through 85th birthday, 85+), which allowed us to program the age-specific death rate and track the aging of cohorts with relative accuracy.

### Results

### Transmissibility of *H. pylori* in 1850

The values of transmissibility most consistent with the endemic pattern of infection were: $\beta_{CC}$ = ***0.000055***, $\beta_{YY}$ = ***0.000011***, and $\beta_{AA}$ = ***0.0000012***. This translated into Potential Transmission Rates (PTR, defined as the average number of secondary infections per unit of time that an infected person would produce in an infection-free population) of $PTR_{CC} = 0.6$ for children (one infected child would be expected to transmit the pathogen to 0.6 susceptible child per year), $PTR_{YY} = 0.25$ for youths, and $PTR_{AA} = 0.15$ for adults. In real populations, these transmission parameters would result in lower incidence rates, since no population is infection free.

### Change in Transmissibility from 1850 to 1995

We estimated the change in transmissibility from 1850 to 1995 that was most consistent with the historical trends of DU and GC and with current ***H. pylori*** prevalence in the United States (Figure 2). The fastest decrease occurred in the latter half of the 19th century. Transmissibility then leveled off at $\beta_{CC} = 0.000042$, $\beta_{YY} = 0.0000044$, and $\beta_{AA} = 0.0000009$. Only a pronounced decrease in $\beta$ at the end of the 19th century could explain the decline in GC incidence and deaths observed. Such a plateau in transmissibility was necessary

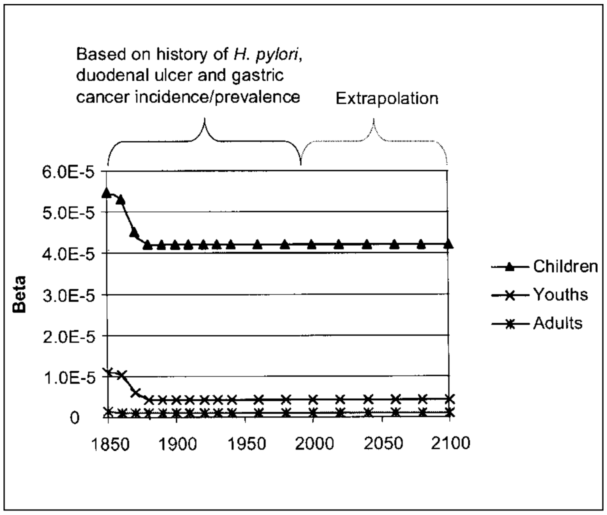

Figure 2. Temporal change in transmission parameters. The transmissibility values in 1850 yielded a pattern of infection similar to that observed in developing countries today (rapid acquisition in younger ages and lower acquisition in older ages). The rapid decline of transmissibility in the latter half of the 19th century is consistent with GC and DU patterns. Although the graph does not show the decrease in $\beta_{AA}$ because of the scale ($\beta_{AA}$ ***is*** much smaller than $\beta_{CC}$ and $\beta_{YY}$), $\beta_{AA}$ also decreased over time. Constant extrapolation of transmissibility assumes no change in standards of living that would affect ***H. pylori*** transmission.

for the prevalence of ***H. pylori*** infection to coincide with current rates. An S-shaped Gompertz curve (33)—in which the decrease is slow initially, accelerates, and slows down again, trending to a stable level—best illustrates the past trends in ***H. pylori*** and associated DU and GC.

### *H. pylori* Prevalence, DU Incidence, and GC Incidence

We simulated the temporal trend of ***H. pylori*** prevalence in the general U.S. population by age category (Figure 3a). Among children, prevalence decreased from 30% in 1850 to 1% by the end of the 20th century; among youths, prevalence decreased from approximately 70% to 5%, and among adults, from approximately 80% to less than 20% in the same period. We compared the simulation outputs with available U.S. data on incidence of GC and DU (Figure 3b). According to the two sources on GC incidence and deaths (25,28), total GC incidence declined from approximately 34 per 100,000 in 1930 to 6.6 per 100,000 in 1995. In comparison, ***H. pylori***-associated GC, estimated from the simulation, decreased from 24 per 100,000 in 1930 to 5.8 per 100,000 in 1995. Kurata and colleagues, based on data from a large health-maintenance organization in southern California from 1977 to 1980, estimated the incidence of DU to be 58 per 100,000 (34). In comparison, our model estimated ***H. pylori***-associated DU incidence to be 47 per 100,000 in 1980.

To extend the simulation through 2100, we assumed that the transmissibility of ***H. pylori*** would remain stable at the level in 1995 (Figure 2) and kept the input parameters constant. ***H. pylori*** prevalence, as well as the associated DU and GC, would continue to decrease in the 21st century. By 2100, the model predicted incidences of 1.3 and 12.2 per 100,000 population for ***H. pylori***-associated DU and GC, respectively, with overall ***H. pylori*** prevalence decreasing to 4.2%.

### Sensitivity Analyses

We tested the model by using different input assumptions. For each change in the input assumptions, we generated curves of ***H. pylori*** prevalence and incidences of ***H. pylori***-associated GC and DU.[1] The most sensitive variables were the proportion of persons with antrum- (vs.

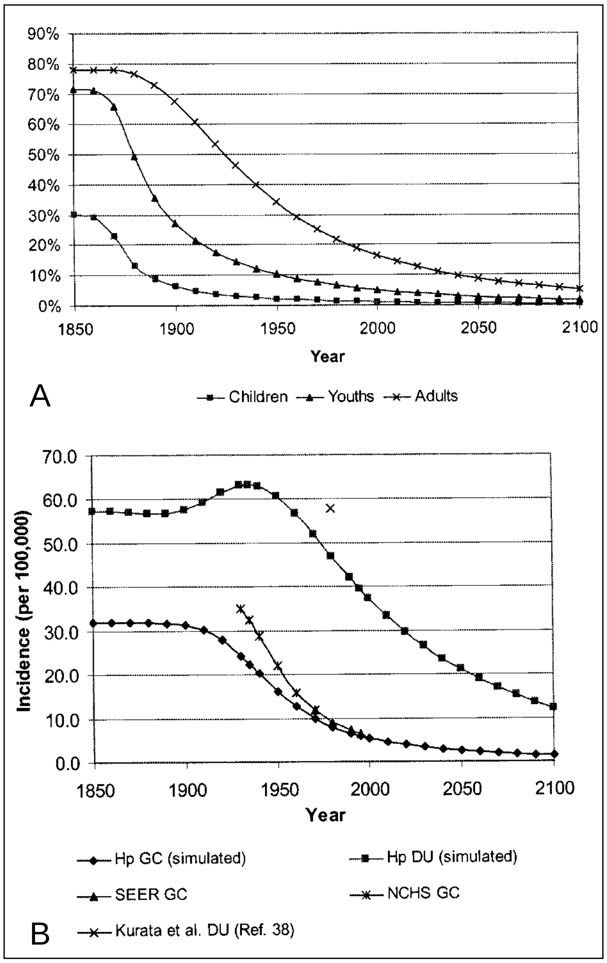

Figure 3. Simulated temporal change in ***Helicobacter pylori*** and associated diseases. a) Prevalence of ***H. pylori*** in the U.S. population by age category (simulation output). b) Incidence of DU and GC per 100,000 U.S. population. "Hp GC" and "Hp DU" are simulation outputs. "SEER GC" represents incidence of GC from all cases, from the Surveillance, Epidemiology, and End Results report (25). "NCHS GC" represents incidence of GC from all cases, derived from the mortality statistics published by the National Center for Health Statistics and the Bureau of the Census (28). "Kurata et al. DU" represents incidence of DU from all cases, based on data from a large health maintenance organization in southern California (34). The simulation model predicted that the prevalence of ***H. pylori***, as well as incidence of ***H. pylori***-associated DU and GC, would continue to decrease in the 21st century.
GC = gastric cancer; DU = duodenal ulcer; NCHS = National Center for Health Statistics; SEER = Surveillance, Epidemiology, and End Results system.

[1]Specific graphs can be made available to readers upon request.

corpus-) predominant gastritis, rate of progression from AG to DU, and rate of progression from CAG to GC. For example, when the rate of progression from CAG to GC was 0.0035, the incidence of ***H. pylori***-associated GC decreased from 6.7 per 100,000 in 1995 to 1.5 per 100,000 in 2100; when the rate was assumed to be 0.0025, the incidence in 1995 was 4.9 per 100,000, decreasing to 1.1 per 100,000 by the end of the 21st century. Overall, we found that ***H. pylori*** prevalence would decrease to <10% in the general U.S. population and the incidence of ***H. pylori***-associated DU and GC would decrease to <20 and 1.5 per 100,000 population, respectively, by the end of the 21st century.

### Discussion

This work provides insights into the intrinsic dynamics of ***H. pylori*** and associated diseases, applying and extending the basic principles of mathematical modeling used extensively in infectious disease analysis. First, we found that in the United States, transmissibility of ***H. pylori*** has already become so low that the incidence and prevalence of the organism will continuously decrease in the foreseeable future, without any targeted intervention. The disappearance of ***H. pylori***, however, would take more than a century without intervention. Second, we found that only a rapid decline in ***H. pylori*** transmissibility during the second half of the 19th century could explain the rapid decrease in GC incidence observed in the 20th century. We tested different curves of decrease in ***H. pylori*** transmissibility and found that a Gompertz curve best fits the past trends of ***H. pylori*** and associated GC and DU. Gompertz curves have been used to describe decline of living organisms, as well as mechanical devices (33). Our findings suggest that those curves could also be used to represent waning of transmissibility (infectivity or transmission potential) of an organism in a given host population.

The decline in transmissibility paralleled the rapid improvement in sanitation and hygiene that occurred in the 19th and early 20th centuries in the United States. In a history of hygiene in the United States, Hoy describes how the United States, once a country with poor sanitary conditions, became one with exemplary cleanliness (35). The author states that "in 1850, cleanliness in the United States, north and south, rural and urban, stood at Third World levels." During the Civil War era, appreciation for cleanliness expanded. Frequent washing of clothes and bathing with soap, expanded availability of clean running water, and development of a sewage network in the late 19th century were among improvements in personal hygiene. Although we cannot rule out other causes, this massive improvement in sanitary and hygienic conditions (better household hygiene and community sanitation projects, including water purification systems), which was responsible for reducing the incidence of infectious diseases such as shigellosis and typhoid, could also explain the decrease in transmissibility of ***H. pylori***.

This model attempted to explain quantitatively how the shift in age of ***H. pylori*** acquisition could have produced the different patterns of DU and GC outcomes. We cannot rule out other factors (such as change in ***H. pylori*** strains), not included in our model, which could have been responsible for the rise and fall of DU and the parallel decrease in GC.

We can indirectly estimate ***H. pylori***–associated GC from National Center for Health Statistics (NCHS) and Surveillance, Epidemiology, and End Results (SEER) curves showing GC incidence from all cases, based on attributable risk (Figure 3b). Since attributable risk for ***H. pylori*** is believed to have decreased because the proportion of proximal GC is higher today than it was at the beginning of the 20th century, the total GC curve underestimates the decline in ***H. pylori***-associated GC. Thus, the incidence of ***H. pylori***-associated GC from our simulation is higher than the one estimated by the method of attributable risk applied to NCHS/SEER data. This discrepancy can be explained by the existence of other factors acting in conjunction with ***H. pylori*** to promote the development of GC. The discrepancy, however, does not affect our conclusions about future decreasing trends in ***H. pylori*** and associated diseases.

In this study, we did not model the demographic changes—such as increase in birth rate and life expectancy—that occurred in the United States from 1850 to the present. Demographic changes affect the epidemiology and disease statistics in many ways. For example, an increase in birth rate would affect population density and crowding, depending on availability of land. Change in the size of a family unit could also affect the transmission dynamics.

The model quickly becomes too complex and intractable. We therefore fixed the population parameters as in other infectious disease transmission models (8,36,37). Because our simulation covers a long time span (1850 to 2100), to compromise between the past, present, and future, we derived the population characteristics from the demographic data of 1950. In sensitivity analyses, we also tested different birth and death rates, but the predictions for future ***H. pylori*** prevalence and incidence of ***H. pylori***-associated DU and GC did not differ significantly from the base case.

In our baseline model, we assumed that 20% of the population would not become infected with ***H. pylori***. This is based on studies from developing countries, where prevalence in older adults is typically 70% to 90%. Given the high rates of transmission in childhood required to account for this high endemicity, the prevalence of nonsusceptible persons (because they are either immune or physically isolated) is critical in explaining why 100% prevalence has not been observed in any population. The model, however, can accommodate a range of values, including 100% prevalence.

We simplified the quantification of transmission parameters by considering only transmission between persons in the same age group. In reality, transmission can also occur between adults and children or adults and youths. However, no data are available on the magnitude of transmission between or among different age groups. Therefore, we also tested the model by using positive numbers for inter-age group transmission, but the predictions for future ***H. pylori*** prevalence and incidence of associated GC and DU did not differ significantly from our base case results.

Given the model estimation that ***H. pylori*** transmissibility has remained relatively constant since the beginning of 20th century, despite many recent developments and changes in lifestyle, we assumed that maintenance of the current ***H. pylori*** transmissibility would be a reasonable extrapolation for the next century. Despite this assumption of constant transmissibility, the model predicted that ***H. pylori*** prevalence, as well as ***H. pylori***-associated DU and GC, would continue to decrease without intervention. Thus, the decreasing trends indicate that transmissibility of ***H. pylori*** has already declined below the threshold value needed to maintain the organism endemic in the population. Without any targeted intervention, however, the disappearance of ***H. pylori*** from the U.S. population will take more than a century.

### Acknowledgments

The authors thank S.L. Hazell, F. Megraud, and M. Blaser for helpful comments and information on the epidemiology and transmission of ***H. pylori***; D. Graham, M. Dixon, J.I. Wyatt, and P. Correa on the natural history of ***H. pylori*** infection; S.M. Blower and M.M. Tanaka on the infectious disease modeling; and P. Glynn on the mathematical formulation.

This study was supported in part by the Centers for Disease Control and Prevention, grant no. 97FEDO9763. Marcia F.T. Rupnow was supported by a scholarship from CNPq Agency, Brazilian Ministry of Education, grant number 20.0724/96-7. Douglas K. Owens is supported by a career development award from the Department of Veterans Affairs Health Services Research and Development Service.

Dr. Rupnow is a senior analyst of strategic modeling with OrthoBiotech, Inc. Her professional interests include mathematical modeling of disease processes and interventions, medical decision analysis, and pharmacoeconomics.

### References

1. Taylor DN, Parsonnet J. Epidemiology and natural history of ***Helicobacter pylori*** infection. In: Blaser MJ, Smith PD, Ravdin JI, Greenberg HB, Guerrant RL, editors. Infections of the gastrointestinal tract. New York: Raven Press; 1995. p. 551-63.
2. NIH Consensus Conference. ***Helicobacter pylori*** in peptic ulcer disease. NIH Consensus Development Panel on ***Helicobacter pylori*** in Peptic Ulcer Disease. JAMA 1994;272:65-9.
3. Telford JL, Ghiara P. Prospects for the development of a vaccine against ***Helicobacter pylori***. Drugs 1996;52:799-804.
4. Lee A. Vaccination against ***Helicobacter pylori***. J Gastroenterol 1996;31 Suppl 9:69-74.
5. Parsonnet J, Blaser MJ, Perez-Perez GI, Hargrett-Bean N, Tauxe RV. Symptoms and risk factors of ***Helicobacter pylori*** infection in a cohort of epidemiologists. Gastroenterology 1992;102:41-6.
6. Banatvala N, Mayo K, Megraud F, Jennings R, Deeks JJ, Feldman RA. The cohort effect and ***Helicobacter pylori***. J Infect Dis 1993;168:219-21.
7. Parsonnet J. The incidence of ***Helicobacter pylori*** infection. Aliment Pharmacol Ther 1995;9 Suppl 2:45-51.
8. Anderson RM, May RM. Infectious diseases of humans: dynamics and control. New York: Oxford University Press; 1991.
9. Kuipers EJ. ***Helicobacter pylori*** and the risk and management of associated diseases: gastritis, ulcer disease, atrophic gastritis and gastric cancer. Aliment Pharmacol Ther 1997;11 Suppl 1:71-88.

10. Graham DY. ***Helicobacter pylori***: its epidemiology and its role in duodenal ulcer disease. J Gastroenterol Hepatol 1991;6:105-13.
11. Schultze V, Hackelsberger A, Günther T, Miehlke S, Roessner A, Malfertheiner P. Differing patterns of ***Helicobacter pylori*** gastritis in patients with duodenal, prepyloric, and gastric ulcer disease. Scand J Gastroenterol 1998;33:137-42.
12. Sonnenberg A. Temporal trends and geographical variations of peptic ulcer disease. Aliment Pharmacol Ther 1995;9 Suppl 2:3-12.
13. Sipponen P, Kekki M, Haapakoski J, Ihamäki T, Siurala M. Gastric cancer risk in chronic atrophic gastritis: statistical calculations of cross-sectional data. Int J Cancer 1985;35:173-7.
14. Sipponen P. ***Helicobacter pylori*** gastritis—epidemiology. J Gastroenterol 1997;32:273-7.
15. Hansson LE, Nyrén O, Hsing AW, Bergström R, Josefsson S, Cho WH, et al. The risk of stomach cancer in patients with gastric or duodenal ulcer disease. N Engl J Med 1996;335:242-9.
16. Blaser MJ, Chyou PH, Nomura A. Age at establishment of ***Helicobacter pylori*** infection and gastric carcinoma, gastric ulcer, and duodenal ulcer risk. Cancer Res 1995;55:562-5.
17. Correa P, Cuello C, Duque E, Burbano LC, Garcia F, Bolanos O, et al. Gastric cancer in Colombia. III. Natural history of precursor lesions. J Natl Cancer Inst 1976;57:1027-35.
18. U.S. National Office of Vital Statistics. Abridged life tables—United States, 1950. Vital Statistics—Special Reports 1953;37:333-43.
19. Gibson C. Population of the 100 largest cities and other urban places in the United States: 1790 to 1990: U.S. Census Bureau, 1998. Available from: URL: http://www.census.gov/population/www/documentation/twps0027.html.
20. Feldman RA, Eccersley AJ, Hardie JM. Epidemiology of ***Helicobacter pylori***: acquisition, transmission, population prevalence and disease-to-infection ratio. British Medical Bulletin 1998;54:39-53.
21. Graham DY. ***Helicobacter pylori*** infection in the pathogenesis of duodenal ulcer and gastric cancer: a model. Gastroenterology 1997;113:1983-91.
22. Valle J, Kekki M, Sipponen P, Ihamäki T, Siurala M. Long-term course and consequences of ***Helicobacter pylori*** gastritis. Results of a 32-year follow-up study. Scand J Gastroenterol 1996;31:546-50.
23. Bonnevie O. The incidence of duodenal ulcer in Copenhagen county. Scand J Gastroenterol 1975;10:385-93.
24. Bonnevie O. The incidence of gastric ulcer in Copenhagen county. Scand J Gastroenterol 1975;10:231-9.
25. SEER Cancer Statistics Review 1973-1995, 1998. Available from: URL: http://www-seer.ims.nci.nih.gov/Publications/CSR7395.
26. Karnes WE, Samloff IM, Siurala M. Positive serum antibody and negative tissue staining for ***Helicobacter pylori*** in subjects with atrophic body gastritis. Gastroenterology 1991;101:167-74.
27. American Cancer Society. Cancer facts and figures—1998. New York: The Society; 1998.
28. Garfinkel L. Cancer statistics and trends. In: Murphy GP, Lawrance W Jr, Lenhard RE Jr, editors. American Cancer Society textbook of clinical oncology. Atlanta: American Cancer Society; 1995. p. 1-9.
29. Susser M. Causes of peptic ulcer. A selective epidemiologic review. Journal of Chronic Diseases 1967;20:435-56.
30. Sonnenberg A. Factors which influence the incidence and course of peptic ulcer. Scand J Gastroenterol Suppl 1988;155:119-40.
31. Sonnenberg A. Geographic and temporal variations in the occurrence of peptic ulcer disease. Scand J Gastroenterol Suppl 1985;110:11-24.
32. Kuipers EJ, Thijs JC, Festen HP. The prevalence of ***Helicobacter pylori*** in peptic ulcer disease. Aliment Pharmacol Ther 1995;9 Suppl 2:59-69.
33. Olshansky SJ, Carnes BA. Ever since Gompertz. Demography 1997;34:1-15.
34. Kurata JH, Honda GD, Frankl H. The incidence of duodenal and gastric ulcers in a large health maintenance organization. Am J Public Health 1985;75:625-9.
35. Hoy S. Chasing dirt. New York: Oxford University Press; 1995.
36. Williams JR, Nokes DJ, Medley GF, Anderson RM. The transmission dynamics of hepatitis B in the UK: a mathematical model for evaluating costs and effectiveness of immunization programmes [published erratum appears in Epidemiol Infect 1996 Oct;117:409]. Epidemiol Infect 1996;116:71-89.
37. Porco TC, Blower SM. Quantifying the intrinsic transmission dynamics of tuberculosis. Theor Popul Biol 1998;54:117-32.

### Appendix
